# Supplementary material for: From sequence to enzyme mechanism using multi-label machine learning
Source: BMC Bioinformatics. 2014 May 19;15:150. doi: 10.1186/1471-2105-15-150 (PMC4229970; doi:10.1186/1471-2105-15-150)
Supplement: Additional file 2 — Java code of ml2db. Additional file ml2db_code.tar.gz contains the Java source code to run the multi-label machine learning experiments and save the results to database. The code’s Javadoc is included. [file 1471-2105-15-150-S2.zip › additional file 2/ml2db/utils/doc/uk/ac/standrews/utils/main/LogUtils.html]

LogUtils


JavaScript is disabled on your browser.


- Overview
- Package
- Class
- Use
- Tree
- Deprecated
- Index
- Help

- Prev Class
- Next Class

- Frames
- No Frames

- All Classes

- Summary:
- Nested |
- Field |
- Constr |
- Method

- Detail:
- Field |
- Constr |
- Method


uk.ac.standrews.utils.main

## Class LogUtils

- java.lang.Object
- - uk.ac.standrews.utils.main.LogUtils

- ---

    

  ```
  public class LogUtils
  extends java.lang.Object
  ```

  Utilities to log error messages

  Version:
  :   27 Feb 2009

  Author:
  :   Luna De Ferrari luna.deferrari-at-ed.ac.uk

- - ### Constructor Summary

    Constructors

    Constructor and Description

    `LogUtils()`
  - ### Method Summary

    Methods

    | Modifier and Type | Method and Description |
    | --- | --- |
    | `static java.lang.String` | `error(java.lang.Object o)` |
    | `static void` | `error(java.lang.Object o, java.lang.String text)` |
    | `static void` | `error(java.lang.String text)` |
    | `static java.lang.String` | `log(java.lang.Object o)` |
    | `static void` | `log(java.lang.Object o, java.lang.String text)` |
    | `static void` | `log(java.lang.String text)` |
    | `static void` | `logAndPrint(java.io.File logFile, java.lang.String text)` |
    | `static void` | `logAndPrint(java.io.File logFile, java.lang.String text, boolean timestamped)` Logs some text to file AND prints it to screen. |
    | `static void` | `logAndPrint(java.lang.String logFilePath, java.lang.String text)` |
    | `static void` | `logAndPrintNoTimestamp(java.io.File logFile, java.lang.String text)` |
    | `static void` | `logMilliseconds(java.lang.String string)` |
    | `static void` | `redirectStdErr(java.io.File file)` Redirect stderr to file |

    - ### Methods inherited from class java.lang.Object

      `equals, getClass, hashCode, notify, notifyAll, toString, wait, wait, wait`

- - ### Constructor Detail


    - #### LogUtils

      ```
      public LogUtils()
      ```
  - ### Method Detail


    - #### error

      ```
      public static java.lang.String error(java.lang.Object o)
      ```


    - #### error

      ```
      public static void error(java.lang.Object o,
               java.lang.String text)
      ```


    - #### error

      ```
      public static void error(java.lang.String text)
      ```


    - #### log

      ```
      public static java.lang.String log(java.lang.Object o)
      ```


    - #### log

      ```
      public static void log(java.lang.Object o,
             java.lang.String text)
      ```


    - #### log

      ```
      public static void log(java.lang.String text)
      ```


    - #### logAndPrint

      ```
      public static void logAndPrint(java.io.File logFile,
                     java.lang.String text)
      ```


    - #### logAndPrint

      ```
      public static void logAndPrint(java.lang.String logFilePath,
                     java.lang.String text)
      ```


    - #### logAndPrint

      ```
      public static void logAndPrint(java.io.File logFile,
                     java.lang.String text,
                     boolean timestamped)
      ```

      Logs some text to file AND prints it to screen. If the file is null, it
      prints to screen only.

      Parameters:
      :   `logFile` - the log file (can be null)
      :   `text` - the message to be logged
      :   `timestamped` - also adds a timestamp to each line


    - #### logAndPrintNoTimestamp

      ```
      public static void logAndPrintNoTimestamp(java.io.File logFile,
                                java.lang.String text)
      ```


    - #### redirectStdErr

      ```
      public static void redirectStdErr(java.io.File file)
      ```

      Redirect stderr to file


    - #### logMilliseconds

      ```
      public static void logMilliseconds(java.lang.String string)
      ```

      Parameters:
      :   `m_string` -


- Overview
- Package
- Class
- Use
- Tree
- Deprecated
- Index
- Help

- Prev Class
- Next Class

- Frames
- No Frames

- All Classes

- Summary:
- Nested |
- Field |
- Constr |
- Method

- Detail:
- Field |
- Constr |
- Method
